# A randomised feasibility study of serial magnetic resonance imaging to reduce treatment times in Charcot neuroarthropathy in people with diabetes (CADOM)

**DOI:** 10.1186/s13047-023-00601-7

**Published:** 2023-01-26

**Authors:** Catherine Gooday, Frances Game, Jim Woodburn, Fiona Poland, Erika Sims, Ketan Dhatariya, Lee Shepstone, Garry Barton, Wendy Hardeman

**Affiliations:** 1 School of Health Sciences, University of East Anglia, Norwich, NR4 7TJ UK; 2 Elsie Bertram Diabetes Centre, Norfolk & Norwich University Hospitals NHS Foundation Trust, Norwich, NR4 7UY UK; 3 Department of Diabetes and Endocrinology, University Hospitals of Derby and Burton NHS Foundation Trust, Derby, DE22 3NE UK; 4 School of Health Sciences and Social Work, Griffith University, Southport, QLD 4222 Australia; 5 Institute for Volunteering Research, Faculty of Medicine and Health Science, University of East Anglia, Norwich, NR4 7TJ UK; 6 Norwich Clinical Trials Unit, Norwich Medical School, University of East Anglia, Norwich, NR4 7TJ UK; 7 Norwich Medical School, University of East Anglia, Norwich, NR4 7TJ UK; 8 School of Health Sciences - Behavioural and Implementation Science Group, Faculty of Medicine and Health Sciences, University of East Anglia, Norwich, NR4 7TJ UK

**Keywords:** Charcot neuroarthropathy, Remission, Diabetes, MRI, Temperature monitoring, X-ray, Feasibility study

## Abstract

**Aim:**

This study aims to explore the feasibility of using serial MRI without contrast in the monitoring of Charcot neuroarthropathy to reduce duration of immobilisation of the foot, in order to decide whether a large-scale trial is warranted.

**Methods:**

A multicentre, randomised, prospective, two arm, open, feasibility study (CADOM) of people with diabetes with a suspected or confirmed diagnosis of Charcot neuroarthropathy. Participants were randomised (1:1) to ‘standard care plus’, including repeated foot temperature measurements and X-rays, or the intervention arm, with additional three-monthly MRI, until remission of Charcot neuroarthropathy or a maximum 12 months (active phase). Participants were then followed-up for a further 6 months, post remission to monitor for relapse of the Charcot neuroarthropathy (follow-up phase). Feasibility outcomes were recruitment, retention, data completeness, adherence to study procedures and safety of the intervention MRI. We also collected clinical efficacy outcomes, this included time in cast/off-loading device which will be the primary outcome of a future definitive trial. Finally, we collected patient reported outcomes, and data on health and social care usage.

**Results:**

One-hundred and five people were assessed for eligibility at five sites. 64/105 potential participants meet the eligibility criteria to participate in the study. Forty-three participants were randomised: 20 to standard care plus and 23 to MRI intervention. The main reason for ineligibility was a previous episode of Charcot neuroarthropathy. Thirteen participants were withdrawn post-randomisation due to an alternative diagnosis being made. Of the remaining 30 participants, 19 achieved remission, 6 had not gone into remission at the end of the 12 month active phase so exited the study. Five participants were lost to follow-up. Of the MRIs that were not disrupted by COVID-19 pandemic 26/31 (84%) were completed. For the visits that were conducted face-to-face, completion rates of patient-reported outcome measures were between 71 and 100%. There were no safety incidents associated with the intervention MRI.

As this was a feasibility study it was not designed to test the effectiveness of serial MRI in diagnosing remission. The time in cast/off-loading device was 235 (±108.3) days for the standard care plus arm compared to 292 (±177.4) days for the intervention arm. There was no statistical difference in the time in cast/off-loading device between the two arms of the study: Hazard Ratio (HR) 0.405 (95% CI 0.140–1.172), *p* = 0.096.

**Discussion:**

The findings support a definitive randomised controlled trial to evaluate the effectiveness of MRI in diagnosing remission in Charcot neuroarthropathy. The rates of recruitment, retention, data, and MRI completeness show that a definitive study is feasible.

**Study registration:**

ISRCTN, 74101606. Registered on 6 November 2017.

**Supplementary Information:**

The online version contains supplementary material available at 10.1186/s13047-023-00601-7.

## Novelty statement/What’s new

- Monitoring and diagnosing remission in Charcot neuroarthropathy are challenging because they rely on techniques which do not accurately reflect disease progression.
- Current evidence suggests Magnetic Resonance Imaging may be superior to current monitoring techniques.
- This study is the first to assess the feasibility of conducting a definitive trial to evaluate the use of serial Magnetic Resonance Imaging (intervention) to reduce treatment times in Charcot neuroarthropathy.
- Despite the COVID-19 pandemic, study recruitment and retention were good.
- The intervention was safe, feasible and acceptable.
- A definitive trial on the use of serial Magnetic Resonance Imaging in monitoring Charcot neuroarthropathy is justified.

## Background

Charcot neuroarthropathy is a relatively rare but serious complication that can affect people with peripheral neuropathy. It is most commonly diagnosed in people with diabetes, usually affecting the foot. There is uncontrolled inflammation, bones become weakened, and this can lead to fractures and joint dislocation.

In 2018 a regional survey of 205,033 people with diabetes in the East Midlands, England reported a point prevalence of 0.04% [1]. Population-based studies have estimated a lifetime cumulative incidence for Charcot neuroarthropathy of 0.4 to 1.3% in people with diabetes, and 13% in those attending diabetic foot specialist clinics [2].

Usual care for people with Charcot neuroarthropathy is to offload pressure and immobilise the foot with a non-removable cast or boot [3]. This aims to stop the inflammatory process, relieve pain, and maintain foot architecture [3]. Studies from the UK and Brazil show a median time to remission of up to 12 months [4–6]. Other studies report shorter times to remission: 3–5 months (US) [7, 8] and 3–6 months (Germany) [9].

Guidelines for Charcot neuroarthropathy management state that off-loading and immobilisation should be continued until the temperature difference between the affected and unaffected foot is < 2 °C, with no further radiological changes on X-ray [10]. However, this recommendation is based on low quality level IV evidence – i.e., case series and expert opinion, rather than higher quality evidence from systematic reviews, randomised controlled trials, case control, or cohort studies [10].

Skin temperature measured with infrared thermography is recommended as a monitoring technique in Charcot neuroarthropathy, as it involves inflammation of the soft tissue and bone [3]. Skin temperature is a proxy measure of inflammation measured over the site of injury and may not reflect the degree of inflammation within affected bones and/or joints. X-rays show damage to the foot skeleton rather than disease activity and are a measure of foot deformity. In contrast, magnetic resonance imaging (MRI) provides detailed pictures of bone and soft tissue structures and can show abnormalities not evident on X-rays [11]. Emerging evidence from case series and observational data suggests that MRI may be useful for monitoring active Charcot neuroarthropathy [12–14], and that MRI findings could be adopted as the criterion standard for assessing disease activity and remission. However, evidence supporting a definitive trial is currently lacking. We conducted a study to assess feasibility outcomes; eligibility, recruitment, retention, withdrawal rates, acceptability of serial MRI for participants, and clinical efficacy outcomes. As this is a feasibility study, we did not investigate the cost effectiveness of the intervention.

## Aims and objectives

This study aims to explore the feasibility of using serial MRI without contrast in the monitoring of Charcot neuroarthropathy to reduce duration of immobilisation of the foot, in order to decide whether a large-scale trial is warranted.

## Methods

Full details of the protocol are published elsewhere [15]. This was a two arm, multicentre, open, randomised controlled feasibility study. Participants were recruited from specialist diabetic foot clinics. The study was divided into two phases. Phase one, the active phase, until the Charcot neuroarthropathy was in remission, or a maximum of 12 months. Phase two, the follow-up phase, for 6 months after the apparent remission of the Charcot neuroarthropathy. The study was approved by East Midlands - Derby Research Ethics, 04/10/2017, ref.: 17/EM/0288. All participants provided written consent.

### Participants – inclusion and exclusion criteria

Participants were aged over 18 years old with diabetes as defined by the World Health Organisation [16] and with a suspected or confirmed diagnosis of Charcot neuroarthropathy. The full inclusion and exclusion criteria is shown in Table 1. We decided to exclude people with a previous history of Charcot neuroarthropathy as new and relapsed cases of Charcot neuroarthropathy may have different healing times. We chose a cut off period of six-months based on the opinion of clinical experts within the trial management team, as we thought that this would ensure that only true new cases of Charcot neuroarthropathy were recruited to the study. As a confirmed diagnosis of Charcot neuroarthropathy can take several weeks, participants were recruited as early as possible to accurately collect the duration of wearing an off-loading device. This was because ‘time in cast/off-loading device ’ is the proposed primary outcome. If the clinical team decided on an alternative diagnosis, the participant exited the study.

**Table 1:** Inclusion and exclusion criteria

Inclusion Criteria	Exclusion Criteria
Participants who are willing and have capacity to give informed consent.	People who have received a transplant and others receiving immunosuppressant therapy or using long-term oral glucocorticoids other than in the routine management of glucocorticoid deficiency. Participants on a low dose of oral glucocorticoids (<10mgs for ≤7 days) are eligible to participate in the study.
People with diabetes as diagnosed by the WHO criteria [16]	Participation in another intervention study on active Charcot.
Age 18 years or over.	Contra-indication for MRI.
New or suspected diagnosis of acute Charcot (no previous incidence of acute Charcot within the last 6 months on the same foot) treated with off-loading.	Treatment for previous suspected Charcot on the same foot in the last 6 months.
Understand written and verbal instructions in English.	Suspected or confirmed bilateral active Charcot at presentation.
	Active osteomyelitis at randomisation.
	Previous contralateral major amputation.
	Inability to have an MRI scan.
	People receiving palliative care.

*Abbreviations*: *MRI* Magnetic Resonance Imaging, *WHO* World Health Organisation

### Randomisation, blinding and data collection

Eligible participants were randomly assigned using a web-based randomisation process on a 1:1 basis to: (a) Immobilisation discontinued on the basis of clinical remission determined by skin temperature measurement, which triggered an MRI (standard care plus) or (b) Standard care plus and additionally the serial use of MRI at 3, 6, 9 and 12 months to identify disease remission and discontinuation of immobilisation (intervention).

### Sample size

As this was a feasibility study a power calculation was not required. An allowance was made for 10–15% of participants to be withdrawn from the study due to an alternative diagnosis. We planned to recruit 60 people with 30 participants per arm, based on recommended sample sizes of between 24 and 50 for a feasibility study [17].

### Study interventions

#### Standard care plus

Participants received usual care for assessment and management of Charcot neuroarthropathy. We standardised assessment of foot temperature to monitor Charcot neuroarthropathy by using the same device for all the measurements taken during the study, the Thermofocus 01500A3® (Tecnimed, Varese, Italy). Every 14 days a research team member measured the temperature of both feet at five different sites. Temperatures were collected four times after removal of the off-loading device: 0, 5, 10, and 15 minutes. It was not possible to control ambient air temperature, in the clinical rooms used in this study. In this feasibility study the first timepoint at which the temperature difference was assessed as ≤2 °C at the site overlying the Charcot neuroarthropathy was used as the marker for remission. At remission participants received a study specific MRI. We used the term ‘standard care plus’ for this arm of the study to highlight differences to usual practice in the protocol for temperature measurement, and the additional study specific MRI at remission.

#### Intervention

In addition to standard care plus, intervention participants received serial MRIs at 3, 6, 9 and 12 months. They did not receive further MRIs once remission was diagnosed. The median time for remission of Charcot neuroarthropathy is reported as between 3 and 12 months. Therefore, we decided that the time to first intervention MRI should reflect the shortest reported median time to remission. As this was a feasibility study, we did not seek to standardise the MRI sequencing protocol.

### Study procedures

The schedule of enrolment, interventions and assessments is shown in Supplementary Fig. 1. Potential participants were approached when attending their regular foot clinic appointment. After giving written informed consent participants attended visits every 14 days until remission. Participants received usual care for Charcot neuroarthropathy regardless of randomisation arm. At the baseline visit participant characteristics, Charcot neuroarthropathy characteristics and type of off-loading device were collected. At each visit foot temperatures were measured, and clinical efficacy outcomes such as new ulceration, foot infection, and amputation (major and minor) were collected. Participants received a patient diary every fortnight to record all health and social care use. The main purpose of the patient diary was to inform how the data on costs and effects would be collected within a definitive trial. We will estimated completion rates and sought to identify big cost drivers, in order to inform this decision. Patient-reported outcome questionnaires EQ-5D-5L (Euroqol 5D), Medical Outcomes Short-Form Health Questionnaire (SF-12) and Hospital Anxiety and Depression Scale (HADS) measuring health-related quality of life, anxiety and depression were collected at enrolment and every 3 months. During the first wave of the UK COVID-19 pandemic (March–August 2020), approval was granted for sites to post questionnaires to participants and be returned to the sponsor, instead of collection during face-to-face study visits. Where research visits were disrupted due to the COVID-19 pandemic, but clinical visits continued, the study sponsor approved the use of information collected from clinical visits.

### Outcomes

We recorded participants’ characteristics; sex, age, diabetes and diabetes related complications, foot complications, stage, and location of the Charcot neuroarthropathy, and type of off-loading device. We measured feasibility, clinical efficacy, and patient reported outcomes. At every visit we issued participants with a diary to collect health and social care usage (Table 2). The proposed primary outcome time in cast/off-loading device was assessed to provide an initial efficacy estimate to inform the sample size of a future definitive study. We also collected data on ulceration, infection, and amputation. The safety of the intervention was assessed. The intervention (MRI) was assessed as low risk and consisted of increased frequency of MRI scans without contrast, so a pragmatic approach to safety reporting was used. MRI scans were performed in NHS hospitals under routine clinical protocols. Adverse safety events resulting from MRI scans were reported by the research teams in line with the Hospital Trust’s clinical incident reporting policy. These were forwarded to the Chief Investigator, for review by the Trial Management Team.

**Table 2:** Feasibility, clinical efficacy, patient reported and health and social care usage

Feasibility outcomes	Clinical efficacy outcomes ***Collected – all study visits***	Patient reported outcomes Collected – baseline, 3 monthly until remission, then at 1 and 6-months post remission
The proportion of patients who meet the eligibility criteria	Time in cast/off-loading device	Health related quality of life measured: • SF-12 [18] • EQ-5D-5L [19], analysed using the crosswalk methodology [20]
The number of eligible participants recruited	Number of new ulcerations on the index foot	HADS [21]
The number of participants in which an alternative diagnosis is made during the active phase of the study	Number of new ulcerations on the index or contralateral foot	VAS
The proportion of participants that withdraw or are lost to follow up. The term ‘withdrawal’ encompasses two potential scenarios: withdrawal due to loss of consent or withdrawal due to death	Number of new infections on the index or contralateral foot	**Data on health and social care usage- Patient Diary**
		• Change in employment • Frequency and the amount of time participants received help with personal and household tasks • Number of all healthcare appointments • Number and severity of falls (Hopkins Fall Grading System) [22]
Statistical parameters of the key outcome measures to inform a sample size calculation for a definitive study	Number of minor and major amputations on the index foot or contralateral foot at the end of the follow up phase of the study	
Safety of the intervention (MRI).	The number of participants in each arm requiring further intervention for Charcot (e.g., further immobilisation) within 6 months of remission	
Data completeness and adherence to study procedures		
Participant acceptability of MRI - the number of MRIs declined or not attended by participants		

*Abbreviations*: *EQ-5D-5L* Euroqol 5D, *HADS* Hospital Anxiety and Depression Scale, *SF-12* Medical Outcomes Short-Form Health Questionnaire, *VAS* Visual analogue scale

In standard care plus, remission was defined as a temperature difference of ≤2 °C between the affected and unaffected foot which was maintained or improved on two separate consecutive occasions for a period of at least 4 weeks [10] or at the discretion of the clinical team when temperature difference was not valid; e.g., in the presence of bilateral foot disease. This triggered a final MRI. In the intervention arm, remission was defined as an absence of sub-chondral bone marrow oedema on MRI. The clinical team will interpret the results of the MRI report to determine remission. Relapse was defined as a reading of ≥2 °C at the site overlying the CN or an adjacent area compared to the contralateral foot maintained for two or more occasions or further changes on imaging.

### Statistical analysis

Descriptive statistics will be used to summarise participants’ baseline characteristics and feasibility outcomes. All analyses will be conducted using a modified Intention-To-Treat approach. Whereby participants that are identified as post-randomisation exclusions (those participants who were identified as having an alternative diagnosis, and therefore failed the inclusion criteria will be excluded from the modified ITT analysis). All participants with a confirmed diagnosis will be included in the analysis. Estimates of outcome variability (e.g., standard deviation) were made with 95% confidence intervals to inform future sample size calculations. The primary efficacy outcome, ‘time in cast/off-loading device’, was analysed using a Cox Proportional Hazards (PH) regression model. Two covariates recorded at baseline were  included in the model: 1) off-loading device, removable or non-removable; and 2) Eichenholtz classification, stage 0 or stage 1 (based on clinical and X-ray findings).

## Results

Five sites in England participated: one in the East of England, two in the East Midlands and two in Yorkshire and Humber. Participant recruitment took place between December 2017 and November 2019. Forty-three participants were randomised, 23 to intervention and 20 to standard care plus.

### Participant characteristics

Baseline characteristics were similar between the two study arms (Table 3). The mean age of participants was 59.1 (±SD 11) years, of whom 29/43 (67%) were men; 34/43 (79%) had type 2 diabetes. Mean diabetes duration was 19 (±SD 11.2) years. Twenty-seven participants, (63%) reported symptoms for > 1-month before a consultation with the specialist multidisciplinary foot team. Common self-reported precipitating factors were concurrent ulceration or a recent trip or fall. The most frequent site for the Charcot neuroarthropathy was the Sanders and Frykberg II (tarso-metatarsal joints). The site of Charcot neuroarthropathy was not reported in 5/30 (17%) of cases. At enrolment 41/43 (95%) had an Eichenholtz Classification stage 0 or I. 21/43 (49%) participants initially received treatment with a non-removable below-knee device, in all cases this was a total contact cast (Table S1).

**Table 3:** Baseline participant characteristics

Baseline participant characteristics	All participants		Confirmed diagnosis of Charcot	
	All randomised participants [***n*** = 43]	Confirmed diagnosis Charcot [***n*** = 30]	Standard care plus (***n*** = 16)	Intervention (***n*** = 14)
**Sociodemographic**				
Men n [%]	29 [67%]	20 [67%]	12 [75%]	8 [57%]
Age (yrs) mean ± SD	59.1 ± 11.2	59.2 ± 10.8	59.2 ± 9.4	51.5 ± 10.8
**Highest education n [%]**	*n* = 37	*n* = 27	*n* = 13	*n* = 14
Left school before 16	4 [11%]	2 [7%]	1 [8%]	1 [7%]
Stayed in school until 16	11 [30%]	8 [30%]	4 [31%]	4 [29%]
Stayed in education until 18	6 [16%]	4 [15%]	3 [23%]	1 [7%]
Vocational/occupational qualification	8 [22%]	7 [30%]	2 [15%]	5 [36%]
Degree	6 [16%]	4 [15%]	2 [15%]	2 [14%]
Masters	1 [3%]	1 [4%]	1 [8%]	0
Doctorate	1 [3%]	1 [4%]	0	1 [7%]
**Diabetes and diabetes related complications**				
Type 2 diabetes n [%]	34 [79%]	22 [73%]	12 [75%]	10 [71%]
Duration of diabetes (yrs) mean ± SD	19 ± 11.2	20.5 ± 11.3	24.6 ± 13	15.8 ± 6.7
HbA1c mmol/mol median [25th–75th IQR]	69 IQR 57–87	77.5 IQR 60–96	73.5 IQR 61–84	77.5 IQR 59–99
eGFR < 60 n [%]	13 [30%]	9 [30%]	3 [19%]	6 [43%]
Type 1 BMI mean ± SD	30.9 ± 6.3	32.1 ± 5.4	33.8 ± 5.9	30.6 ± 5.1
Type 2 BMI mean ± SD	32.5 ± 7.0	32.1 ± 6.5	32.2 ± 7.7	32.2 ± 5.2
Cerebrovascular events n [%]	4 [9%]	2 [7%]	1 [6%]	1 [7%]
Cardiovascular events n [%]	10 [23%]	6 [20%]	2 [13%]	4 [29%]
Nephropathy n [%]	11 [26%]	9 [30%]	2 [13%]	7 [50%]
Retinopathy n [%]	18 [42%]	14 [47%]	10 [63%]	4 [29%]
**Palpation foot pulses n [%]**				
No foot pulses palpable	4 [9%]	3 [10%]	1 [6%]	2 [14%]
One-foot pulse palpable	1 [2%]	0	0	0
Two-foot pulses palpable	38 [88%]	27 [90%]	15 [94%]	12 [86%]
**Ankle Brachial Index n [%]**	*n* = 41	*n* = 28	*n* = 15	*n* = 12
Ankle Brachial Index 0.5–0.79	1 [2%]	1 [4%]	1 [4%]	0
Ankle Brachial Index 0.8–0.99	4 [10%]	3 [11%]	0	3 [25%]
Ankle Brachial Index 1.0–1.4	31 [76%]	22 [79%]	13 [81%]	9 [75%]
Ankle Brachial Index > 1.4	5 [12%]	2 [7%]	2 [13%]	0
**Monofilament perception n [%]**				
-ve at 3/3 sites	33 [77%]	24 [80%]	13 [81%]	11 [79%]
-ve at 2/3 sites	4 [9%]	0	0	0
-ve at 1/3 sites	1 [2%]	2 [7%]	1 [6%]	1 [7%]
+ve at all sites n [%]	5 [12%]	4 [13%]	2 [13%]	2 [14%]
**Mean vibration perception at hallux**	*n* = 29	*n* = 22	*n* = 11	*n* = 11
Hallux (≥25 V)	23 [79%]	19 [86%]	10 [91%]	9 [82%]
**Previous or current foot complications n [%]**				
Previous minor amputation index foot	7 [16%]	5 [17%]	4 [25%]	1 [7%]
Previous minor amputation contralateral foot	5 [12%]	4 [13%]	3 [19%]	1 [7%]
History of previous Charcot either foot	6 [14%]	6 [20%]	3 [19%]	3 [21%]
Ulceration at enrolment on index foot	12 [30%]	7 [23%]	5 [31%]	2 [14%]
Ulceration at enrolment on contralateral foot	1 [2%]	0	0	0

*Abbreviations*: *BMI* Body mass index, *eGFR* Estimated Glomerular Filtration rate, ml/min, *HbA1c* Glycated haemoglobin (A1c), mmol/mol

### Feasibility outcomes

#### Eligibility

Participants were approached during routine foot clinic visits. 64/105 (61%) potential participants met the eligibility criteria. Nine participants had multiple reasons for exclusion (Table S2). The main reason was a history of Charcot neuroarthropathy within the last 6 months. Of the potentially eligible participants 43/64 (67%) agreed to study participation.

#### Participant retention

**Fig. 1:**
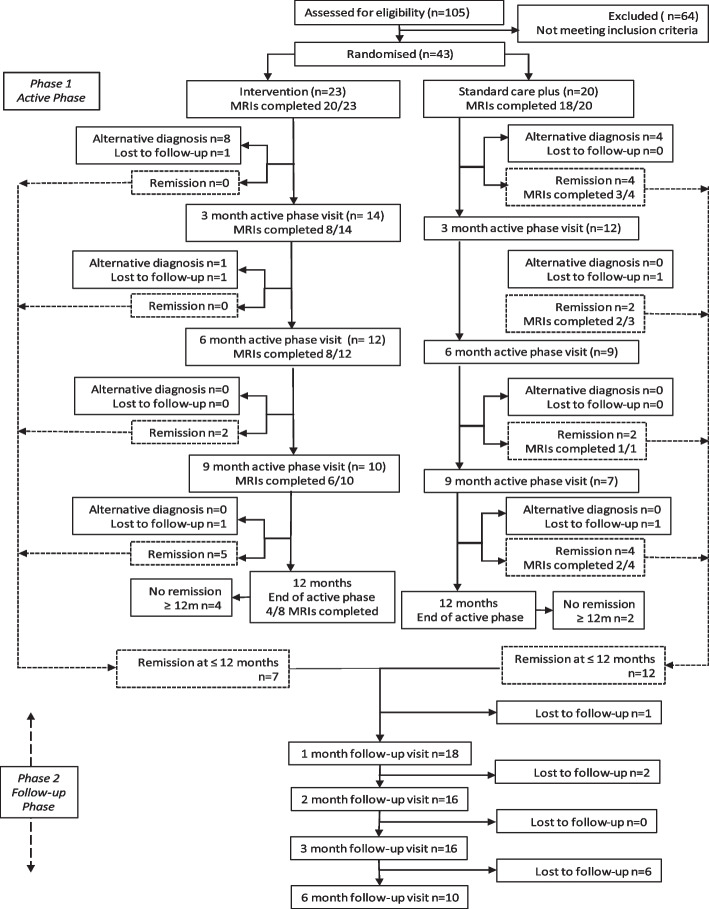
Consort diagram

Figure 1 shows the CONSORT diagram. 30/43 (70%) participants received a confirmed Charcot neuroarthropathy diagnosis, the remaining 13 (30%) exited the study due to an alternative diagnosis (3 stress fracture, 2 osteoarthritis, 2 infection, 1 soft tissue injury, 5 not reported). Of the 30 participants with a confirmed Charcot neuroarthropathy diagnosis, 19 (63%) went into remission, and 5 were lost to follow-up (2 relocated, 2 due to COVID-19 and 1 unknown). Six (20.0%) did not achieve remission at the end of the 12-month active phase. They did not progress into the follow-up phase but were included in analyses. During the six-month follow-up phase, two participants experienced a relapse, one in each study arm.

### Adherence to study procedures

During the active phase, 469/497 (94%) of visits not disrupted by COVID-19 pandemic were completed. 438/497 (88%) of study visits were completed in the one-week timeframe window (Table S3). 79 visits were partially or completely disrupted by COVID-19.

For participants with a confirmed Charcot neuroarthropathy diagnosis, 26/31 (84%) of MRIs that were not disrupted by COVID-19 pandemic were completed. It is not known why the remaining five did not go ahead. A further twelve were missed due to changed research radiology priorities during COVID-19 pandemic (Table S4). Of the MRIs that went ahead, 16/26 (62%) were undertaken within the two-week window; the median time for the remaining ten was 20 days either side of the 14-day window (range − 30 to + 48 days). Nine non-study MRIs were completed in each study arm; no data are available on which foot they concerned.

### Clinical efficacy, safety, and participant acceptability outcomes

All 30 participants with a confirmed diagnosis of Charcot neuroarthropathy were included in the Cox regression analysis hazard model. In this feasibility study there was no statistical difference in the time in cast/off-loading device between the two arms of the study: Hazard Ratio (HR) 0.405 (95% CI 0.140–1.172), *p* = 0.096 (Table 4). As this was a feasibility study it was not designed to test the effectiveness of serial MRI in diagnosing remission. The time in cast/off-loading device was 235 (±108.3) days for the standard care plus arm compared to 292 (±177.4) days for the intervention arm. Time in cast/off-loading device will be the proposed primary outcome in a future trial. Participants who were provided with a non-removable device went into remission sooner than those treated with a removable device: HR 0.285 (95% CI 0.107–0.758), *p* = 0.012. The Eichenholtz classification at baseline did not affect time to remission: HR 1.083 (95% CI 0.349–3.362), *p* = 0.890.

**Table 4:** Results from Cox regression analysis on time in cast/off-loading device (days)

Enrolment ***n*** = 30	Covariates	Time in cast/off-loading device (days)		***P***-value	Hazard Ratio	95.0% Confidence Interval	
		Mean	SD			Lower	Upper
Randomisation arm	Intervention	292.6	±108.3	0.096	0.405	0.140	1.172
	Standard care plus	235.2	±117.4				
Off-loading device	Non-removable	198.6	±117.8	0.012	0.285	0.107	0.758
	Removable	325.3	±70.3				
Eichenholtz stage	Stage 0	267.5	±108.5	0.890	1.083	0.349	3.362
	Stage I	252.4	±130.4				

One participant in the standard care plus arm was admitted to hospital because of foot complications; no minor or major amputations were reported. Thirty-eight ulcerations were recorded on 19 participants during the active phase, of which 12 were attributed to the off-loading device. Participants reported 84 falls, six required outpatient medical attention, and a further four hospital admissions. No safety events associated with the intervention (MRI) were reported. No participants declined an MRI or failed to attend their MRI imaging appointment.

### Data completion and patient reported outcome results

For the visits that were conducted face-to-face, completion rate of patient-reported outcome measures were between 71 and 100% (Table S5 and S6). Half of the participants reported anxiety and depression scores higher than normal levels. The majority of participants scored (well) below normal levels for the physical component score, and just under half scored (well) below normal for the mental component score [18]. Nearly all participants reported pain and problems with mobility and completing usual activities, and often relied on support from family and friends. The EQ-5D index calculated using the crosswalk mechanism and self-rated health status was lower than that for aged-matched population normals [23].

### Patient diary – health and social care usage

A number of data points in the patient diary were not completed, and in places we were unable to distinguish between truly missing data or zero values that were not reported. Five participants reported job changes during the study. During the active phase, participants reported a mean of 18.4 healthcare related visits per participant. 62% of appointments concerned the foot. Nineteen participants reported they needed help from family or friends to complete activities of daily living on 599 occasions ranging from 5 to 900 minutes per week. Common tasks requiring help were shopping, cleaning, and bathing.

## Discussion

This feasibility study recruited 43 participants with a suspected or confirmed Charcot neuroarthropathy diagnosis. Despite not achieving the recruitment target of 60 participants, and study interruptions due to the COVID-19 pandemic, our sample size was sufficiently large enough to draw conclusions about the feasibility of a definitive trial.

Our participants were representative of the wider Charcot neuroarthropathy population, with more men, being in their fifth decade, and diabetes duration >10 years [24]. In this study the most common site for the Charcot neuroarthropathy was Sanders and Frykberg II (tarsometatarsal joints), this is consistent with other studies [4]. The main reason for ineligibility was a previous Charcot neuroarthropathy within the last 6 months. We argue that it is not possible to reduce this as it may affect the results with the inclusion of relapsed Charcot neuroarthropathy, which may have a different time to remission than new cases.

Two-thirds of eligible participants agreed to take part. We recruited similar participant numbers to observational studies on monitoring techniques [6, 12, 25] and recent randomised controlled trials on pharmacological treatment of Charcot neuroarthropathy [26, 27]. More participants than anticipated were withdrawn from our study due to an alternative diagnosis. This reflects the difficulty in diagnosing Charcot neuroarthropathy at Eichenholtz stage 0. During the active phase the attrition rate was 12% which is within acceptable limits and does not affect the results of this study [28].

Two-thirds of participants had an Eichenholtz Classification stage 0 at baseline with no changes on X-ray. This highlights the need to use MRI as an adjunct to X-rays in Charcot neuroarthropathy diagnosis and monitoring. Excluding the effect of COVID-19 pandemic on MRIs, 84% of intervention MRIs were completed, and no safety incidents reported. No participants declined an MRI. This supports the feasibility, safety, and participants’ acceptability of serial MRIs.

Participants experienced multiple episodes of concurrent ulceration and infection. This highlights the previously recognised limitations of using infra-red thermography to monitor Charcot neuroarthropathy. If similar findings were replicated in a definitive trial this further justifies the need to use MRI as a monitoring technique for Charcot neuroarthropathy.

The signal from this feasibility study does suggest that MRI may extend the time in cast/off-loading device. However, this feasibility study was not powered to detect a difference between the two arms of the study. As this was not an effectiveness study, the observed trend may change in an adequately powered definitive trial. The COVID-19 pandemic changed clinical practice, and reduced adherence to study procedures. We do not know how these changes affected the proposed primary outcome. Therefore, at this stage uncertainty remains and the trial is warranted as the sample size cannot exclude the possibility of a worthwhile effect.

Disruption caused by the COVID-19 pandemic resulted in participants being transferred from non-removable to removable devices, reduced data collection, and intervention completeness. This means that the planned modified ITT Cox regression analysis cannot be relied upon to calculate an accurate simple size. We plan to re-analyse the data using a per protocol analysis to mitigate for the impact of the COVID-19 pandemic. We will also review the literature and use a focus group to work with patient and public involvement, healthcare professionals, and statisticians to agree the minimally important clinical difference for a reduction in ‘time in cast’. This information will then be used in a future sample size calculation.

The results related to patient-reported outcomes need to be interpreted with caution: this is a feasibility study not powered to detect between-group or longitudinal differences. We showed that receiving treatment for Charcot neuroarthropathy has physical, emotional, and socioeconomic ramifications and are results are consistent with others [29]. The minimum clinically important difference which people perceive as beneficial and should result in a change in management for EQ-5D has been estimated to be 0.03 [30]. In this study the change in the EQ-5D index score from baseline to remission showed an improvement in health status of + 0.155 (Table S6). This increase shows that using serial MRI to diagnose Charcot neuroarthropathy remission has potential to be cost-effective. We also showed the important and unrecognised burden of informal care for people with Charcot neuroarthropathy. In a definitive study we will seek to capture the cost of this informal care and include this in a cost effectiveness analysis.

### Strengths and limitations

The strength of this study is the recruitment of study participants who are representative of the wider population of people living with Charcot neuroarthropathy. The study was embedded within clinical practice and study visits were designed to align with pre-existing clinical pathways for Charcot neuroarthropathy. The clinical team took responsibility for both clinical care and the research. This approach increased the number of people who were willing to participate in the research and contributed to the high retention levels observed in this study.

One potential limitation of this study was that MRI sequencing protocol was not standardised; however, this study did not seek to provide a definitive answer as to the efficacy of using serial MRI to diagnose remission so was not necessary. The non-study MRIs that were completed may have contaminated the results, diluting the relationship between intervention and outcome. We do not know whether these MRIs reflected a change in practice due to study participation.

The COVID-19 pandemic reduced data and intervention completeness and the clinical efficacy and patient reported outcomes that were collected. During the first wave of the UK COVID-19 pandemic, some hospitals involved in the study stopped or restricted research radiology imaging, reducing the number of completed study MRIs. This may have resulted in either an underestimation or overestimation of the time in cast/off-loading device. Our participants were considered a ‘clinically vulnerable’ group and advised by the UK government to only attend essential clinical visits which excluded study visits. Consequently, the end point time in cast/off-loading device and other outcomes were not always collected. Some clinical teams transferred participants from non-removable to removable offloading devices to reduce the number of follow-up appointments. Wearing removable devices is associated with increased time to remission [4]. This may have increased the end point time in cast/off-loading device. We do not have data on whether the two arms of the study were equally affected by this.

### Implications for a definitive study

A definitive study on serial MRI in people with Charcot neuroarthropathy attending multidisciplinary foot clinics is justified and feasible, based on recruitment and retention rates, and acceptability of the intervention and study visit schedule to participants.

In a future study randomisation should be stratified for the type of off-loading device. Normal plain X-rays, widespread inflammation, and participants awaiting their baseline MRI meant that it was sometimes difficult for investigators to classify the location of the Charcot neuroarthropathy at baseline. To address this missing data, we will modify the data collection tool to ask investigators to confirm or record the location of the Charcot neuroarthropathy at baseline and the 3 month follow-up visit. We found low levels of data completeness in the patient diary. Therefore, in a future study we will modify the patient diary and collect a more complete picture of participants’ costs. The diary will be co-produced with patient and public involvement to ensure the diary is relevant to and reflects the priorities of people with Charcot neuroarthropathy and other key stakeholders.

Prior to any future definitive trial, we will undertake a Delphi consensus study to optimise and standardise the intervention serial MRI. Finally, we will seek to capture the reasons for any non-study MRIs.

A continuation of the COVID-19 pandemic or another pandemic may disrupt a future study. To prepare for the possibility of this we will seek to maximise data collection through additional participant reported outcomes. This could include the type of off-loading device being used, self-monitored foot temperatures when participants are transferred from non-removable to removable devices to reduce the number of hospital appointments they need to attend, and outcomes such as ulceration and infection.

If a future definitive study proves that MRI is a more effective way to monitor Charcot neuroarthropathy, then this could save healthcare providers money. People would regain their independence and go back to work sooner.

## Conclusion

This feasibility study showed that a future definitive trial to evaluate the effectiveness of MRI to identify disease remission in CN is warranted, feasible and acceptable, to potential participants, healthcare, and research professionals. We recruited 67% of potentially eligible participants and 88% of these completed the active phase of the study. Excluding the disruption caused by the COVID-19 pandemic 94% of study visits and 84% of the intervention MRIs were completed. The absence of any safety incidents supports the feasibility and safety of serial MRIs as the intervention. The results of this study will inform the design, of a full randomised controlled trial.

## Supplementary Information

**Additional file 1: Supplementary Table 1.** Baseline Charcot neuroarthropathy characteristics and type of offloading provided at enrolment.

**Additional file 2: Supplementary Table 2.** Main reasons for ineligibility.

**Additional file 3: Supplementary Table 3.** Adherence - Numbers of study visit completed.

**Additional file 4: Supplementary Table 4.** Adherence - Numbers of intervention MRIs completed.

**Additional file 5: Supplementary Table 5.** Results for the completion rates of patient reported outcome measures.

**Additional file 6: Supplementary Table 6.** Results for the patient reported outcome measures.

**Additional file 7: Supplementary Fig. 1.** Schedule of enrolment, interventions, and assessments.

## Data Availability

The datasets generated and/or analysed during the current study will be available from the corresponding author on reasonable request, provided appropriate credit is attributed to the original authors and the data source.

## Abbreviations

ABPI: Ankle brachial pressure index
BMI: Body mass index
CADOM: Charcot neuroarthropathy diagnostic outcome measures
CI: Confidence Interval
CN: Charcot neuroarthropathy
eGFR: Estimated Glomerular Filtration rate, ml/min
EQ-5D-5L: Euroqol 5D
F: Follow up visit
FU: Follow up phase
HADS: Hospital Anxiety and Depression Scale
HbA1c: Glycated haemoglobin (A1c), mmol/mol
HR: Hazard Ratio
MRI: Magnetic Resonance Imaging
NHS: National Health Service
R: Remission
SD: Standard deviation
SF-12: Medical Outcomes Short-Form Health Questionnaire
VAS: Visual analogue scale
WHO: World Health Organisation

## References

[1] Metcalf L, Musgrove M, Bentley J, Berrington R, Bunting D, Mousley M (2018). Prevalence of active Charcot disease in the east midlands of England. Diabetic Med.

[2] Armstrong D, Todd W, Lavery L, Harkless L, Bushman T (1997). The natural history of acute Charcot’s arthropathy in a diabetic foot speciality clinic. Diabet Med.

[3] National Institute for health and care excellence. Diabetic foot problems : prevention and management. NG19. 2015:1–49 Available from: https://www.nice.org.uk/guidance/ng19. Cited 2021 Dec 30. 26741017

[4] Game F, Catlow R, Jones G, Edmonds M, Jude E, Rayman G (2012). Audit of acute charcot’s disease in the UK: the cduk study. Diabetologia.

[5] Stark C, Murray T, Gooday C, Nunney I, Hutchinson R, Loveday D (2016). 5 year retrospective follow-up of new cases of Charcot neuroarthropathy—a single centre experience. Foot Ankle Surg.

[6] Moura-Neto A, Fernandes T, Zantut-Wittmann D, Trevisan R, Sakaki M, Santos A (2012). Charcot foot: skin temperature as a good clinical parameter for predicting disease outcome. Diabetes Res Clin Pract.

[7] Pinzur M, Lio T, Posner M (2006). Treatment of Eichenholtz stage 1 Charcot foot arthropathy with a weight-bearing total contact cast. Foot Ankle Int.

[8] de Souza L (2008). Charcot arthropathy and immobilization in a weight-bearing total contact cast. J Bone Joint Surg.

[9] Chantelau E, Kimmerle R, Poll LW (2007). Nonoperative treatment of neuro-Osteoarthropathy of the foot: do we need new criteria?. Clin Podiatr Med Surg.

[10] Milne T, Rogers J, Kinnear E, Martin H, Lazzarini P, Quinton T (2013). Developing an evidence-based clinical pathway for the assessment, diagnosis and management of acute Charcot neuro-arthropathy: a systematic review. J Foot Ankle Res.

[11] Chantelau E, Richter A (2013). The acute diabetic Charcot foot managed on the basis of magnetic resonance imaging - a review of 71 cases. Swiss Med Wkly.

[12] Zampa V, Bargellini I, Rizzo L, Turini F, Ortori S, Piaggesi A (2011). Role of dynamic MRI in the follow-up of acute Charcot foot in patients with diabetes mellitus. Skeletal Radiol.

[13] Chantelau E, Antoniou S, Zweck B, Haage P (2018). Follow up of MRI bone marrow edema in the treated diabetic Charcot foot–a review of patient charts. Diabet Foot Ankle.

[14] Schlossbauer T, Mioc T, Sommerey S, Kessler S, Reiser M, Pfeifer KJ (2008). Magnetic resonance imaging in early stage Charcot arthropathy – correlation of imaging findings and clinical symptoms. Eur J Med Res.

[15] Gooday C, Game F, Woodburn J, Poland F, Sims E, Dhatariya K (2020). A randomised feasibility study of serial magnetic resonance imaging to reduce treatment times in Charcot neuroarthropathy in people with diabetes (CADOM): a protocol. Pilot Feasibility Stud.

[16] World Health Organisation (2011). Use of glycated haemoglobin (HbA1c) in the diagnosis of diabetes mellitus.

[17] Sim J, Lewis M (2012). The size of a pilot study for a clinical trial should be calculated in relation to considerations of precision and efficiency. J Clin Epidemiol.

[18] Ware J, Kosinski M, Keller S (1996). A 12-item short-form health Survey : construction of scales and preliminary tests of reliability and validity. Med Care.

[19] Herdman M, Gudex C, Lloyd A, Janssen M, Kind P, Parkin D (2011). Development and preliminary testing of the new five-level version of EQ-5D (EQ-5D-5L). Qual Life Res.

[20] Van Hout B, Janssen MF, Feng YS, Kohlmann T, Busschbach J, Golicki D (2012). Interim scoring for the EQ-5D-5L: mapping the EQ-5D-5L to EQ-5D-3L value sets. Value Health.

[21] Zigmond A, Snaith R (1983). The hospital anxiety and depression scale. Acta Psychiatr Scand.

[22] Davalos-Bichara M, Lin F, Carey J, Walston J, Fairman J, Schubert M (2013). Development and validation of a falls grading scale. J Geriatr Phys Ther.

[23] Kind P, Hardman G, Macran S (1999). UK population norms for EQ-5D. Discussion paper.

[24] Petrova N, Foster A, Edmonds M (2004). Difference in presentation of Charcot Osteoarthropathy in type 1 compared with type 2 diabetes. Diabetes Care.

[25] Wu T, Chen PY, Chen CH, Wang CL (2012). Doppler spectrum analysis: a potentially useful diagnostic tool for planning the treatment of patients with Charcot arthropathy of the foot? Journal of bone and joint. Surgery.

[26] Petrova N, Donaldson N, Bates M, Tang W, Jemmott T, Morris V (2021). Effect of recombinant human parathyroid hormone (1-84) on resolution of active Charcot neuro-osteoarthropathy in diabetes: a randomized, double-blind, placebo-controlled study. Diabetes Care.

[27] Das L, Bhansali A, Prakash M, Jude E, Rastogi A (2019). Effect of methylprednisolone or zoledronic acid on resolution of active charcot neuroarthropathy in diabetes: a randomized, double-blind, placebo-controlled study. Diabetes Care.

[28] Schulz K, Grimes D (2002). Sample size slippages in randomised trials: exclusions and the lost and wayward. Lancet.

[29] Raspovic KM, Wukich DK (2014). Self-reported quality of life in patients with diabetes: a comparison of patients with and without charcot neuroarthropathy. Foot Ankle Int.

[30] Coretti S, Ruggeri M, McNamee P (2014). The minimum clinically important difference for EQ-5D index: a critical review. Expert Rev Pharmacoecon Outcomes Res.

