# Supplementary material for: A randomised feasibility study of serial magnetic resonance imaging to reduce treatment times in Charcot neuroarthropathy in people with diabetes (CADOM)
Source: J Foot Ankle Res. 2023 Jan 26;16:2. doi: 10.1186/s13047-023-00601-7 (PMC9878485; doi:10.1186/s13047-023-00601-7)
Supplement: Supplementary file 1 — Additional file 1: Supplementary Table 1. Baseline Charcot neuroarthropathy characteristics and type of offloading provided at enrolment. [file 13047_2023_601_MOESM1_ESM.docx]

Supplementary table 1 - Baseline Charcot neuroarthropathy characteristics and type of offloading provided at enrolment

| **Charcot characteristics** | **All participants** | | **Confirmed diagnosis of Charcot** | |
| --- | --- | --- | --- | --- |
| **Characteristics** | **All randomised participants**  **[n=43]** | **Confirmed diagnosis Charcot**  **[n=30]** | **Standard care plus**  **(n=16)** | **Intervention**  **(n=14)** |
| **Location of Charcot n [%]** | | | | |
| Right foot | 23 [54%] | 17 [57%] | 9 [[56%] | 8 [57%] |
| Left foot | 20 [47%] | 13 [43%] | 7 [44%] | 6 [43%] |
| **Duration of signs and symptoms of inflammation prior to diagnosis n [%]** | | | | |
| 0-2 weeks | 4 [9%] | 3 [10%] | 3 [19%] | 0 |
| 2-4 weeks | 12 [30%] | 6 [20%] | 1 [6%] | 5 [36%] |
| >1 month | 20 [47%] | 17 [57%] | 10 [63%] | 7 [50%] |
| >3 months | 6 [14%] | 3 [10%] | 1 [6%] | 2 [14%] |
| > 6 months | 1 [2%] | 1 [3%] | 1 [6%] | 0 |
| **Potential precipitating factors ≤6 months prior to Charcot** (participants could have multiple precipitating factors) | | | | |
| Trip/fall | 10 | 6 | 1 | 5 |
| Trauma | 9 | 7 | 4 | 3 |
| Limb/foot surgery | 2 | 1 | 1 | 0 |
| Ulceration | 12 | 7 | 4 | 3 |
| Other suspected cause Charcot | 3 | 1 | 1 | 0 |
| **Sanders and Frykberg Classification** (participants could have multiple sites in the foot at randomisation) | | | | |
| I | 11 | 7 | 4 | 3 |
| II | 23 | 17 | 7 | 10 |
| III | 8 | 7 | 1 | 6 |
| IV | 5 | 4 | 1 | 3 |
| V | 0 | 0 | 0 | 0 |
| Unknown | 8 | 5 | 5 | 0 |
| **Eichenholtz classification n [%]** | | | | |
| Stage 0 - Prodromal | 29 [67%] | 19 [63%] | 12 [75%] | 7 [50%] |
| Stage i – Developmental | 12 [28%] | 11 [37%] | 4 [25%] | 7 [50%] |
| Stage ii - Coalescence | 2 [5%] | 0 | 0 | 0 |
| Stage iii - Reconstruction | 0 | 0 | 0 | 0 |
| **Off-loading at baseline n [%]** | | | | |
| Non-removable off-loading device | 21 [49%] | 15 [50%] | 9 [56%] | 6 [43%] |
| Removable off-loading device | 21 [49%] | 15 [30%] | 7 [25%] | 8 [36%] |
| Bespoke Footwear | 1 [2%] | 0 | 0 | 0 |
| **Additional walking aids provide n [%]** | | | | |
| Crutches/walking sticks | 15 [35%] | 10 [33%] | 7 [44%] | 3 [21%] |
| Wheelchair | 1 [2%] | 1 [3%] | 0 | 1 [7%] |
