# Supplementary material for: A randomised feasibility study of serial magnetic resonance imaging to reduce treatment times in Charcot neuroarthropathy in people with diabetes (CADOM)
Source: J Foot Ankle Res. 2023 Jan 26;16:2. doi: 10.1186/s13047-023-00601-7 (PMC9878485; doi:10.1186/s13047-023-00601-7)
Supplement: Supplementary file 2 — Additional file 2: Supplementary Table 2. Main reasons for ineligibility. [file 13047_2023_601_MOESM2_ESM.docx]

Supplementary table 2 - Main reasons for ineligibility

| Main reasons for ineligibility ^*^ | Number of participants excluded |
| --- | --- |
| Participants who are unwilling and/or do not have capacity to give informed consent. | 21 |
| Absence of new or suspected diagnosis of acute Charcot (no previous incidence of acute Charcot within the last 6 months on the same foot) treated with off-loading | 21 |
| Active osteomyelitis at randomisation | 8 |
| Contra-indication for MRI | 8 |
| Treatment for previous suspected Charcot on the same foot in the last 6 months | 7 |
| People who have received a transplant and others receiving immunosuppressant therapy or using long term oral glucocorticoids other than in the routine management of glucocorticoid deficiency. People on a low dose of oral glucocorticoids (<10mgs for ≤7 days) are eligible to participate in the study. | 6 |
| Suspected or confirmed bilateral active Charcot at presentation | 5 |
| People without diabetes as defined by the WHO criteria | 3 |
| People receiving palliative care | 1 |
| Previous contralateral major amputation | 1 |
| Participation in another intervention study on active Charcot | 1 |
| Unable to understand written and verbal instructions in English | 0 |
| Aged <18 years | 0 |

^*^Some participants did not meet more than one eligibility criteria

Abbreviations

MRI Magnetic Resonance Imaging

WHO World Health Organisation
