# Supplementary material for: A randomised feasibility study of serial magnetic resonance imaging to reduce treatment times in Charcot neuroarthropathy in people with diabetes (CADOM)
Source: J Foot Ankle Res. 2023 Jan 26;16:2. doi: 10.1186/s13047-023-00601-7 (PMC9878485; doi:10.1186/s13047-023-00601-7)
Supplement: Supplementary file 3 — Additional file 3: Supplementary Table 3. Adherence - Numbers of study visit completed. [file 13047_2023_601_MOESM3_ESM.docx]

Supplementary table 3 – Adherence - Numbers of study visit completed

| **Visit outcome**^*^ | **Active phase**  **n=576** | **Follow-up phase**  **n=80** |
| --- | --- | --- |
| Number of completed visits | 469 [81%] | 49 [61%] |
| Number of visits completed within timeframe window^**^ | 438 [87%] | 36 [60%] |
| Number of partially completed^***^ | 34 [6%] | 11 [14%] |
| Number of missed visits | 28 [5%] | 1 [1.%] |
| Number of missed visits (due to COVID-19) | 45 [8%] | 19 [24%] |

^*^ For those with a confirmed diagnosis of Charcot neuroarthropathy

^**^ The study protocol allowed for a one-week window to complete a visit either side of the actual visit due date

^***^A partially completed visit occurred where study visits were disrupted due to the COVID-19 pandemic, but clinical visits continued, and information collected from clinical visits was used for the study
