# Supplementary material for: A randomised feasibility study of serial magnetic resonance imaging to reduce treatment times in Charcot neuroarthropathy in people with diabetes (CADOM)
Source: J Foot Ankle Res. 2023 Jan 26;16:2. doi: 10.1186/s13047-023-00601-7 (PMC9878485; doi:10.1186/s13047-023-00601-7)
Supplement: Supplementary file 4 — Additional file 4: Supplementary Table 4. Adherence - Numbers of intervention MRIs completed. [file 13047_2023_601_MOESM4_ESM.docx]

Supplementary table 4 – Adherence - Numbers of intervention MRIs completed

| **MRI outcome** | **Active phase^*^** | | | | |
| --- | --- | --- | --- | --- | --- |
|  | **0** | **3m** | **6m** | **9m** | **12m** |
| Total completed [%] | 13/14 [93%] | 8/13 [62%] | 8/12 [67%] | 6/10 [60%] | 4/8 [50%] |
| Missed MRI [%] | 1/14 [7%] | 4/13 [31%] | 0 | 1/10 [10%] | 0 |
| Missed (due to COVID-19)[%] | 0 | 1/13 [8%] | 4/12 [33%] | 3/10 [30%] | 4/8 [50%] |
| Completed in timeframe window [%]^**^ | NA | 6/8 [75%] | 4/8 [50%] | 4/6 [67%] | 2/4 [50%] |

^*^Participants randomised to serial MRIs did not undergo a further MRI once remission had been diagnosed. Therefore, the total number of MRIs decreased over the 12 months.

^**^ The study protocol allowed for a two-week window to complete the MRI either side of the actual MRI due date

Abbreviations

MRI Magnetic Resonance Imaging
