# Supplementary material for: A randomised feasibility study of serial magnetic resonance imaging to reduce treatment times in Charcot neuroarthropathy in people with diabetes (CADOM)
Source: J Foot Ankle Res. 2023 Jan 26;16:2. doi: 10.1186/s13047-023-00601-7 (PMC9878485; doi:10.1186/s13047-023-00601-7)
Supplement: Supplementary file 5 — Additional file 5: Supplementary Table 5. Results for the completion rates of patient reported outcome measures. [file 13047_2023_601_MOESM5_ESM.docx]

Supplementary table 5 – Results for the completion rates of patient reported outcome measures.

|  | **Active phase (until remission or maximum 12 months) ^*^** | | | | | **Follow-up phase ^**^** | |
| --- | --- | --- | --- | --- | --- | --- | --- |
|  | **0** | **3m** | **6m** | **9m** | **12m** | **1m FU** | **6m FU** |
| Visits completed | 30 | 26 | 18 | 14 | 7 | 14 | 10 |
| **VAS – pain** | | | | | | | |
| Completed | 30 [100%] | 23 [88%] | 18 [100%] | 11 [79%] | 5 [71%] | 13 [93%] | 9 [90%] |
| Partially completed | 0 | 0 | 0 | 0 | 0 | 0 | 0 |
| Missed at visit | 0 | 3 [12%] | 0 | 3 [21%] | 2 [29%] | 1 [7%] | 1 [10%] |
| **HADS – Anxiety** | | | | | | | |
| Completed | 26 [87%] | 21 [81%] | 18 [100%] | 10 [71%] | 7 [100%] | 12 [86%] | 9 [90%] |
| Partially completed | 0 | 1 [4%] | 0 | 0 | 0 | 0 | 0 |
| Missed during visit | 4 [13%] | 4 [15%] | 0 | 4 [29%] | 0 | 2 [14%] | 1 [10%] |
| **HADS – Depression** | | | | | | | |
| Completed | 26 [87%] | 22[85%] | 17 [94%] | 10 [71%] | 7 [100%] | 13 [93%] | 9 [90%] |
| Partially completed | 0 | 0 | 1 [6%] | 0 | 0 | 0 | 0 |
| Missed during visit | 4 [13%] | 4 [15%] | 0 | 4 [29%] | 0 | 1 [7%] | 1 [10%] |
| **EQ-5D-5L index** | | | | | | | |
| Completed | 30 [100%] | 23 [88%] | 18 [100%] | 10 [71%] | 5 [71%] | 13 [93%] | 9 [90%] |
| Partially completed | 0 | 1 [4%] | 0 | 1 [7%] | 0 | 0 | 0 |
| Missed during visit | 0 | 2 [8%] | 0 | 3 [21%] | 2 [29%] | 1 [7%] | 1 [10%] |
| **SF-12** | | | | | | | |
| Completed | 29 [97%] | 23 [88%] | 17 [94%] | 11 [79%] | 6 [86%] | 13 [93%] | 9 [90%] |
| Partially completed | 1 [3%] | 1 [4%] | 1 [6%] | 0 | 1 [14%] | 0 | 1 [10%] |
| Missed during visit | 0 | 2 [8%] | 0 | 3 [21%] | 0 | 1 [7%] | 0 |

^*^Participants transferred from the active phase to the follow-up phase of the study once the Charcot neuroarthropathy was assessed as in remission. Therefore, the total number of participants decreases over the 12-month active phase.

^**^Only participants who achieved remission within the 12-month active phase of the study transferred into the follow-up phase of the study.

Abbreviations

EQ-5D-5L Euroqol 5D

FU Follow up phase

HADS Hospital Anxiety and Depression Scale

SF-12 Medical Outcomes Short-Form Health Questionnaire

VAS Visual analogue scale
