# Supplementary material for: A randomised feasibility study of serial magnetic resonance imaging to reduce treatment times in Charcot neuroarthropathy in people with diabetes (CADOM)
Source: J Foot Ankle Res. 2023 Jan 26;16:2. doi: 10.1186/s13047-023-00601-7 (PMC9878485; doi:10.1186/s13047-023-00601-7)
Supplement: Supplementary file 6 — Additional file 6: Supplementary Table 6. Results for the patient reported outcome measures. [file 13047_2023_601_MOESM6_ESM.docx]

Supplementary table 6 – Results for the patient reported outcome measures.

|  | **Active phase (until remission or maximum 12 months) ^*^** | | | | | **Follow-up phase^**^** | |
| --- | --- | --- | --- | --- | --- | --- | --- |
|  | **0** | **3m** | **6m** | **9m** | **12m** | **1m FU** | **6m FU** |
| **VAS pain N=** | **30** | **23** | **18** | **11** | **5** | **13** | **9** |
| mean  ±SD | 18.97 ±29.41 | 20.70 ±26.02 | 13.94 ±19.32 | 12.73 ±19.76 | 15.60 ±25.58 | 15.92±  23.69 | 21.89 ±23.28 |
| **Hospital Anxiety and Depression Scale (HADS)** | | | | | | | |
| **HADS – Anxiety N=** | **26** | **21** | **18** | **10** | **7** | **12** | **9** |
| mean  ±SD | 7.50 ±4.15 | 7.71 ±5.02 | 6.89 ±5.41 | 7.00 ±4.57 | 6.57 ±4.58 | 4.25  ±3.62 | 3.78 ±3.70 |
| Normal score ≤7 n [%] | 13 [50%] | 10 [48%] | 9 [50%] | 6 [60%] | 4 [57%] | 9 [75%] | 7 [78%] |
| Borderline risk n [%] | 7 [27%] | 5 [27%] | 5 [28%] | 2 [20%] | 1 [14%] | 0 [%] | 1 [11%] |
| Intermediate risk n [%] | 6 [23%] | 6 [23%] | 4 [22%] | 2 [20%] | 2 [29%] | 3 [25%] | 1 [11%] |
| **HADS – Depression n=** | **26** | **22** | **17** | **10** | **7** | **13** | **9** |
| mean  ±SD | 6.69 ±4.27 | 7.32 ±5.09 | 6.53 ±5.44 | 8.30 ±4.37 | 7.57 ±6.00 | 4.54  ±4.05 | 5.00 ±4.47 |
| Normal score ≤7 n [%] | 16 [62%] | 14 [64%] | 10 [59%] | 5 [50%] | 3 [43%] | 9 [69%] | 7 [78%] |
| Borderline risk n [%] | 5 [19%] | 2 [9%] | 3 [18%] | 3 [30%] | 2 [29%] | 3 [23%] | 0 |
| Intermediate risk n [%] | 5 [19%] | 6 [27%] | 4 [24%] | 2 [20%] | 2 [29%] | 1 [8%] | 2 [22%] |
| **EQ-5D-5L** | | | | | | | |
| **Mobility n =** | **30** | **24** | **18** | **11** | **5** | **13** | **9** |
| No problems n [%] | 1 [3%] | 3 [13%] | 1 [6%] | 0 | 1 [20%] | 2 [15%] | 0 |
| Some problems n [%] | 29 [97%] | 21 [88%] | 17 [94%] | 11 [100%] | 4 [8O%] | 11[85%] | 9 [100%] |
| **Self-care n =** | **30** | **23** | **18** | **11** | **5** | **13** | **9** |
| No problems n [%] | 15 [50%] | 7 [30%] | 7 [39%] | 3 [27%] | 2 [40%] | 7 [54%] | 5 [56%] |
| Some problems n [%] | 15 [50%] | 16 [70%] | 11 [61%] | 8 [73%] | 3[60%] | 6 [46%] | 4 [44%] |
| **Usual activities n =** | **30** | **24** | **18** | **10** | **5** | **13** | **9** |
| No problems n [%] | 2 [7%] | 2 [8%] | 1 [6%] | 0 | 1 [20%] | 2 [15%] | 0 |
| Some problems n [%] | 28 [93%] | 22 [92%] | 17 [94%] | 10 [100%] | 4 [80%] | 11 [85%] | 9 [100%] |
| **Pain/discomfort n =** | **30** | **24** | **18** | **11** | **5** | **13** | **9** |
| No problem n [%] | 6 [20%] | 6 [25%] | 4 [22%] | 3 [27%] | 2 [40%] | 8 [62%] | 3 [33%] |
| Some problems n [%] | 24 [80%] | 18 [75%] | 14 [78%] | 8 [73%] | 3 [60%] | 5 [38%] | 6 [67%] |
| **Anxiety/depression n =** | **30** | **24** | **18** | **11** | **5** | **13** | **9** |
| No problems n [%] | 11 [37%] | 10 [42%] | 7 [39%] | 4 [36%] | 2 [40%] | 8 [62%] | 6 [67%] |
| Some problems n [%] | 19 [63%] | 14 [58%] | 11 [61%] | 7 [64%] | 3 [60%] | 5 [38%] | 3 [33%] |
| **Index value n=** | **30** | **23** | **18** | **10** | **5** | **13** | **9** |
| Mean  ±SD | 0.525 ±0.23 | 0.565 ±0.26 | 0.531 ±0.24 | 0.562 ±0.23 | 0.597 ±0.32 | 0.68  ±0.19 | 0.628  ±0.17 |
| **Health today n=** | **29** | **24** | **18** | **11** | **5** | **13** | **9** |
| Mean  ±SD | 62.83 ±21.03 | 66.79 ±22.48 | 64.89 ±24.83 | 77.18 ±13.39 | 71.00 ±28.15 | 77.77 ±14.35 | 67.22 ±18.22 |
| **SF-12 N=** | **29** | **23** | **17** | **11** | **6** | **13** | **9** |
| Physical component score (PCS) mean ±SD | 37.42 ±7.18 | 38.44 ±7.43 | 37.25 ±7.34 | 39.56 ±5.43 | 40.48 ±9.0 | 39.71 ±10.77 | 34.47 ±8.06 |
| Same or better n [%] | 5 [17%] | 5 [22%] | 1 [6%] | 2 [18%] | 2 [33%] | 4 [31%] | 2 [22%] |
| Below n [%] | 4 [14%] | 2 [9%] | 6 [35%] | 4 [36%] | 1 [17%] | 2 [15%] | 1 [11%] |
| Well below n [%] | 20 [69%] | 16 [70%] | 10 [59%] | 5 [45%] | 3 [50%] | 7 [54%] | 6 [67%] |
| Mental component score (MCS) mean ±SD | 45.08 ±11.58 | 46.44 ±12.6 | 45.61 ±13.57 | 43.02 ±12.91 | 41.04 ±13.05 | 52.49 ±11.54 | 35.92 ±10.77 |
| Same or better n [%] | 15 [52%] | 15 [65%] | 10 [59%] | 6 [55%] | 2 [33%] | 9 [69%] | 6 [67%] |
| Below n [%] | 5 [17%] | 1 [4%] | 0 | 2 [18%] | 0 | 2 [15%] | 1 [11%] |
| Well below n [%] | 9 [31%] | 7 [30%] | 7 [41%] | 3 [27%] | 4 [67%] | 2 [15%] | 2 [22%] |

^*^Participants transferred from the active phase to the follow-up phase of the study once the Charcot neuroarthropathy was assessed as in remission. Therefore, the total number of participants decreases over the 12-month active phase.

^**^Only participants who achieved remission within the 12-month active phase of the study transferred into the follow-up phase of the study.

Abbreviations

EQ-5D-5L Euroqol 5D

FU Follow up phase

HADS Hospital Anxiety and Depression Scale

SD Standard deviation

SF-12 Medical Outcomes Short-Form Health Questionnaire

VAS Visual analogue scale
