# Supplementary material for: A randomised feasibility study of serial magnetic resonance imaging to reduce treatment times in Charcot neuroarthropathy in people with diabetes (CADOM)
Source: J Foot Ankle Res. 2023 Jan 26;16:2. doi: 10.1186/s13047-023-00601-7 (PMC9878485; doi:10.1186/s13047-023-00601-7)
Supplement: Supplementary file 7 — Additional file 7: Supplementary Fig. 1. Schedule of enrolment, interventions, and assessments. [file 13047_2023_601_MOESM7_ESM.docx]

Supplementary Figure 1 - Schedule of enrolment, interventions, and assessments

|  |  | Active phase* (maximum 12 months) | | | | | | | | | R | **Follow up phase | | | |
| --- | --- | --- | --- | --- | --- | --- | --- | --- | --- | --- | --- | --- | --- | --- | --- |
| Visit Number |  | 0 |  | 6 |  | 11 |  | 18 |  | 26 |  | F1 | F2 | F3 | F4 |
| Month |  | 0 |  | 3 |  | 6 |  | 9 |  | 12 |  | 1 | 2 | 3 | 6 |
| Enrolment | | | | | | | | | | | | | | | |
| Information sheet | * |  |  |  |  |  |  |  |  |  |  |  |  |  |  |
| Consent |  | * |  |  |  |  |  |  |  |  |  |  |  |  |  |
| Randomisation |  | * |  |  |  |  |  |  |  |  |  |  |  |  |  |
| Participant characteristics | | | | | | | | | | | | | | | |
| Medical history |  | * |  |  |  |  |  |  |  |  |  |  |  |  |  |
| HbA1c & eGFR |  | * |  |  |  |  |  |  |  |  |  |  |  |  |  |
| Foot surgical history |  | * |  |  |  |  |  |  |  |  |  |  |  |  |  |
| Medications |  | * |  |  |  |  |  |  |  |  |  |  |  |  |  |
| Classification CN *** |  | * |  |  |  |  |  |  |  |  |  |  |  |  |  |
| Foot assessment | | | | | | | | | | | | | | | |
| Foot pulses |  | * |  |  |  |  |  |  |  |  |  |  |  |  |  |
| ABPI |  | * |  |  |  |  |  |  |  |  |  |  |  |  |  |
| 10g monofilament |  | * |  |  |  |  |  |  |  |  |  |  |  |  |  |
| Neurotheisometer |  | * |  |  |  |  |  |  |  |  |  |  |  |  |  |
| Foot temperatures |  | * | * | * | * | * | * | * | * | * | * | * | * | * | * |
| Treatment | | | | | | | | | | | | | | | |
| Off-loading/footwear |  | * | * | * | * | * | * | * | * | * | * | * | * | * | * |
| Interventions | | | | | | | | | | | | | | | |
| MRI (standard care plus) |  |  |  |  |  |  |  |  |  |  | * |  |  |  |  |
| Serial MRI (intervention) |  |  |  | * |  | * |  | * |  | * |  |  |  |  |  |
| Clinical outcomes | | | | | | | | | | | | | | | |
| Ulceration |  | * | * | * | * | * | * | * | * | * | * | * | * | * | * |
| Infection |  | * | * | * | * | * | * | * | * | * | * | * | * | * | * |
| Amputation |  | * | * | * | * | * | * | * | * | * | * | * | * | * | * |
| Falls |  | * | * | * | * | * | * | * | * | * | * | * | * | * | * |
| BMI |  | * |  | * |  | * |  | * |  | * | * |  |  |  | * |
| X-ray |  |  |  |  |  |  |  |  |  |  |  |  |  |  | * |
| Patient reported outcomes | | | | | | | | | | | | | | | |
| VAS - pain |  | * |  | * |  | * |  | * |  | * |  | * |  |  | * |
| HADS |  | * |  | * |  | * |  | * |  | * |  | * |  |  | * |
| EQ-5D-5L |  | * |  | * |  | * |  | * |  | * |  | * |  |  | * |
| SF-12 |  | * |  | * |  | * |  | * |  | * |  | * |  |  | * |
| Health economic outcomes | | | | | | | | | | | | | | | |
| Issue patient diary |  | * | * | * | * | * | * | * | * | * |  |  |  |  |  |
| Collect patient diary |  |  | * | * | * | * | * | * | * | * | * |  |  |  |  |
| Qualitative research |  |  |  |  |  |  |  |  |  |  |  |  |  |  |  |
| Interview |  |  |  |  |  |  |  |  |  |  |  |  |  |  |  |
